# A light-driven burst of hydroxyl radicals dominates oxidation chemistry in newly activated cloud droplets

**DOI:** 10.1126/sciadv.aav7689

**Published:** 2019-05-01

**Authors:** Suzanne E. Paulson, Peter J. Gallimore, Xiaobi M. Kuang, Jie Rou Chen, Markus Kalberer, David H. Gonzalez

**Affiliations:** 1 Department of Atmospheric and Oceanic Sciences, University of California at Los Angeles, Los Angeles, CA 90095-1565, USA.; 2 Department of Chemistry, University of Cambridge, Lensfield Road, Cambridge CB2 1EW, UK.; 3 Department of Environmental Sciences, University of Basel, Klingelbergstrasse 27, 4056 Basel, Switzerland.

## Abstract

Aerosol particles and their interactions with clouds are one of the most uncertain aspects of the climate system. Aerosol processing by clouds contributes to this uncertainty, altering size distributions, chemical composition, and radiative properties. Many changes are limited by the availability of hydroxyl radicals in the droplets. We suggest an unrecognized potentially substantial source of OH formation in cloud droplets. During the first few minutes following cloud droplet formation, the material in aerosols produces a near-UV light–dependent burst of hydroxyl radicals, resulting in concentrations of 0.1 to 3.5 micromolar aqueous OH ([OH]_aq_). The source of this burst is previously unrecognized chemistry between iron(II) and peracids. The contribution of the “OH burst” to total OH in droplets varies widely, but it ranges up to a factor of 5 larger than previously known sources. Thus, this new process will substantially enhance the impact of clouds on aerosol properties.

## Teaser

Freshly formed cloud droplets produce a burst of highly reactive radicals, which increases their importance in the climate system.

## INTRODUCTION

Aerosols, fog, and clouds have major direct and indirect roles in both climate and air quality. Organic aerosols make up the largest fraction of aerosol mass, with more than half coming from secondary sources (*1*). They remain a significant source of uncertainty, as their mass fractions and degree of oxidation are significantly underpredicted by current models (*2*). Chemistry in cloud water, driven by hydroxyl radicals, is well known to contribute to the production of secondary organic aerosol (SOA), substantially increasing aerosol mass concentrations and changing aerosol size distributions and other properties of the aerosol once the cloud reevaporates (*3*–*7*). Aqueous OH radicals in cloud droplets are a key but uncertain species in determining global sulfate production and resulting direct and indirect radiative effects (*8*–*11*). OH mediates aqueous oxidation of key climate-relevant gases such as methane sulfonic acid and dimethlyl sulfoxide, intermediate products from dimethlysulfide (DMS) oxidation (*9*). Globally, DMS has been estimated to contribute around 16% of sulfate, 18% of sulfate direct radiative forcing, and more than half of the incremental indirect radiative forcing because it is emitted into the clean marine background (*10*). Recently, Chen *et al*. (*11*) concluded that aqueous OH concentrations are the largest source of uncertainty in the process (*11*).

Several sources of OH in cloud and fog drops have been discussed extensively in the literature. (i) Uptake of OH from the gas phase is a process that is thought to be the dominant source of OH radicals in drops under many conditions. For a globally averaged gas-phase OH concentration ([OH]_g_) of 1.1 × 10^6^ molecules cm^−3^ (*12*), this suggests a (maximum) rate of ~2 × 10^−9^ M s^−1^ for a 10-μm-diameter droplet (section S1), although [OH]_g_ varies by a factor of ~100 throughout the diurnal cycle (*13*). Chemical sources within the drops can provide additional OH radicals. These sources include (ii) the breakdown of hydrogen peroxide (H_2_O_2_) catalyzed by transition metals, primarily iron (the Fenton reaction, R1)

$$\text{Fe}(\text{II})+H_{2}O_{2}\to \text{Fe}(\text{III})+\text{OH}+{\text{OH}}^{-}$$ (R1)

(iii) the “photo-Fenton” reaction, in which reaction R1 is promoted by the rapid photoreduction of Fe(III) to Fe(II), followed by the Fenton reaction (*14*); and direct photolysis of (iv) H_2_O_2_ (*15*), (v) iron hydroxides (*16*), (vi) nitrate (*15*), and (vii) nitrite (*15*).

Several studies have explored OH formation from authentic cloud water samples transported to a laboratory and exposed to simulated sunlight and have found overall production rates of (0.03 to 3) × 10^−10^ M s^−1^ during the first 2 hours (*17*, *18*), i.e., significantly slower than the abovementioned OH uptake from the gas phase. No measurable activity in the absence of light was found in these experiments. Instead of investigating the formation of hydroxyl radicals in authentic cloud water, we simulate chemistry in newly formed cloud/fog droplets by adding water to ambient aerosol samples at aerosol/water (dilution) ratios in the range of cloud droplets, usually within 2 hours of their collection. We then illuminate the samples and quantify [OH]_aq_ formation in the solutions.

## RESULTS

**Fig. 1:**
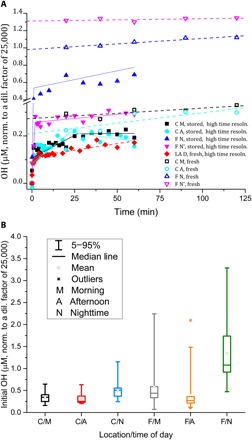
Hydroxyl radical formation by ambient aerosols in water at pH 3.5 normalized to a v:v dilution factor of 25,000. (**A**) OH formation versus time for selected samples. LA, West Los Angeles; C, Claremont; F, Fresno; M, morning; A, afternoon, N, night; D, daytime. Filled symbols/solid lines indicate stored samples; open symbols/dashed lines indicate fresh samples. High time resolution data are fitted with stepwise linear functions. Almost all OH is formed within the first few minutes after the aerosol is brought into contact with water, simulating the cloud droplet nucleation process. (**B**) Hydroxyl radical formation within the first few minutes (see text) for samples collected in Claremont (urban site in summer) and Fresno (urban site in agricultural area in winter; site has notable contribution of biomass burning aerosol during night and early morning), morning (7 a.m. to 1 p.m.), afternoon (1 p.m. to 6 p.m.), and overnight (6 p.m. to 7 a.m.).

When particles are first diluted with water and illuminated with near–ultraviolet (UV) light, a large spike of OH production is observed (Fig. 1A). Stored samples have lower activity than they did when they were fresh, registering about 50 ± 40% of the initial burst (see also Fig. 1A), indicating a limited lifetime of the OH precursors. OH formation was not detected in dark conditions, which suggests a process driven by UV light. Figure 1A shows the evolution of OH formation for the first 60 to 120 min of extraction for four fresh and three stored samples from Fresno, Claremont, and West Los Angeles. Each measurement was made by exposing the sample to 9 s of 315- to 325-nm light, recording a measurement, and repeating twice in rapid succession for a total of 27 s of light exposure. In all cases, the three measurements were not significantly different from one another, indicating that the initial 9 s of light (comparable to the solar flux, as discussed in section S3) was sufficient to photolyze all available OH-producing chromophores. The rapid increase stops abruptly after 2 to 3 min and is followed by a slower, usually linear phase of OH formation. The slower phase of OH production is in the range expected from processes (ii) to (vii) above and is not explored further here. Our kinetic data do not have sufficient time resolution to determine the initial rate of OH formation well, but the rate of formation of OH radicals in the initial burst has a lower limit of ~1 × 10^−9^ to 30 × 10^−9^ M s^−1^, rates that produce ~0.1 to 3.6 μM OH in 2 min (Fig. 1B and see also section S3).

Figure 1B shows interquartile plots of OH formation rates for the initial burst of OH radicals for the Claremont and Fresno samples for morning, afternoon, and overnight, analyzed immediately after collection. These sites were chosen for their different source characteristics (see Methods) and imply that the ability of particles to produce an OH burst will be widespread across many urban and rural atmospheric conditions. The initial burst of OH production produced an average of 0.51 ± 0.4 and 1.6 ± 1.3 μM OH for Claremont and Fresno, respectively, with maxima up to 3.5 μM OH. The high levels of activity observed in the overnight and, to a lesser degree, morning samples collected in Fresno appear to be associated with the substantial contribution of biomass burning aerosol (section S1) and/or accumulation of photolabile OH precursors. Humic-like substances often associated with biomass burning have been shown to increase reaction rates of Fe(II) redox reactions, leading to OH production, including R1 and the reduction of molecular oxygen

$$\text{Fe}(\text{II})+O_{2}\to \text{Fe}(\text{III})+{O_{2}}^{.-}$$ (R2)

by factors of 200 or more (*19*), supporting our observation that nighttime samples show higher OH production. OH formation for other samples was reasonably well correlated to mass. Mass-normalized OH formation is very weakly negatively correlated to the concentration (i.e., positively correlated with the dilution factor) for some samples, with no correlation for others (figs. S3 and S4). This relative lack of concentration dependence implies that a reasonable representation of the short-lived burst of the process might be added to models without detailed consideration of particle/droplet dimensions.

### Source of hydroxyl radical burst

The hydroxyl radical burst is not explained by known pathways to OH formation in cloud drops [processes (i) to (vii) above], as their kinetics are all about one to three orders of magnitude too slow. In laboratory air, OH and H_2_O_2_ are low and thus cannot explain the observed OH levels. H_2_O_2_ measured in the aerosol extraction solutions was also low, i.e., below 2 nM at reaction times of a few minutes, which is the time scale of the observed OH burst (section S5).

**Fig. 2:**
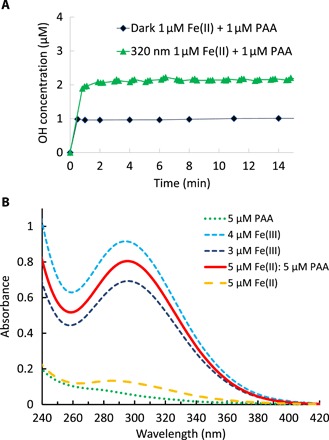
OH formation and speciation in the PAA – Fe(II) system. (**A**) OH concentration versus time by mixtures of PAA and Fe(II) in dark or light (320 nm). Data are corrected for the sum of Fe(II)- and PAA-only controls. The reaction kinetics observed in the laboratory system closely mimics the OH formation kinetics observed for the field samples shown in Fig. 1A. (**B**) Absorbance of 5 μM each of PAA (5 μM, green), Fe(III) from FeCl_3_ at 3 and 4 μM, Fe(II) from FeSO_4_ (5 μM, yellow), and mixtures of 1:1 Fe(II) (FeSO_4_) + PAA (5 μM each) (red line) in aqueous solution adjusted to pH 3.5. After reacting for a few minutes, the 5 μM 1:1 mixture of Fe(II) and PAA results in an absorption spectrum identical to Fe(III), indicating the fast conversion of Fe(II) to Fe(III) and consistent with OH formation burst observed in Fig. 1A and panel (A).

We can produce very similar behavior to that observed in the field samples from mixtures of peracetic acid (PAA) and Fe(II) illuminated with near-UV light (Fig. 2A). The reaction of PAA results in a rapid burst of OH production within the first minute in both light and dark; exposure to 320-nm light approximately doubles the magnitude of the initial burst. This phenomenon requires both PAA and Fe(II) in the solution: OH formation in the dark is below our detection limit (~20 nM) in blank experiments at pH 3.5, which omit PAA, Fe(II), or both. Corresponding blank experiments in the light show only slightly higher OH formation with a small intercept (usually 65 to 90 nM), followed by a slow, small linear increase, the largest of which is for Fe(II) solutions consisting of only Fe(II) and the OH probe in pH 3.5 solution. This was used as the blank for the data shown in Fig. 2A. In the dark and at 1:1 μM Fe:PAA, OH formation is approximately stoichiometric (Fig. 2A); 1 μM OH is formed from Fe(II) and PAA each at 1 μM.

The detailed reaction mechanism of OH formation in the dark Fe-PAA reaction is unknown, but it is likely analogous to the Fenton reaction (R1), where PAA replaces H_2_O_2_

$$\text{Fe}(\text{II})+{\text{CH}}_{3}C(O)\text{OOH}\;(\text{PAA})\to \text{Fe}(\text{III})+\text{OH}+{\text{CH}}_{3}C(O)O^{-}$$ (R3)

The analogous reaction for R3 between free Fe(II) and H_2_O_2_ (R1) is fairly slow [55 M^−1^; (*20*)]; however, several studies have shown enhancements to this rate by several orders of magnitude (2 to 3+) due to iron chelators (*19*, *21*) and aqueous surface reactions (*22*). Because our experiments were performed by adding water to particles on a filter, surface reactions are not likely to play a major role; their role in cloud droplets is unknown.

We have carefully considered if the terephthalate probe for OH might respond to other reaction products than OH in this system, and find it very unlikely (section S7). The OH formation is dependent on Fe(II) and PAA concentrations, but at high PAA concentrations, OH formation yield decreases, suggesting that iron-organic complexes may form at higher concentrations that lower OH reaction yields (section S8). However, substantially higher OH yields for UV exposure conditions (compared to dark) are maintained for all investigated Fe(II)/PAA concentration ratios (fig. S6). The concentration-dependent behavior is generally consistent with the observed weak relationship between OH formation, with increasing dilution for some of the field samples (fig. S4).

The higher OH yield in the presence of UV light might be due to a photo-Fenton–like reaction (R4), where Fe(III) and organic ligands, such as acetate, which is a product of the initial Fenton-like reaction (R3), form a complex leading to the reduction of Fe and the formation of an organic radical that will form OH.

$$\text{Fe}(\text{III})+{\text{CH}}_{3}C(O)O-+h\upsilon \to \text{Fe}(\text{II})+{\text{CH}}_{3}C(O)O\to \text{OH radical formation}$$ (R4)

We also tested *t*-butyl hydroperoxide; this does not generate an OH burst, suggesting that only a subset of organic peroxides contributes to the observed burst in field samples.

Figure 2B shows 240- to 420-nm absorption spectra for Fe(II), Fe(III), PAA, and mixtures of PAA and Fe(II) in concentrations between 3 and 5 μM, in pH 3.5 solution. All spectra were measured at 2 ± 0.5 min after mixing in the dark. Consistent with earlier studies, PAA and Fe(II) have weak absorptions in the region, while Fe(III) absorptions are much stronger (*23*, *24*). Subtraction of the Fe(III) spectrum from the Fe(II)/PAA mixture shows that the two are not differentiable, and indicates rapid conversion of Fe(II) to Fe(III) in the presence of PAA, consistent with the fast OH production in Fig. 2A and reaction R3. This very rapid reaction between Fe(II) and PAA is a previously unknown reaction.

## DISCUSSION

We therefore hypothesize that the large OH “burst” observed in our ambient samples is derived from organic peroxide decomposition in the presence of Fe(II) and potentially other transition metals during simulated cloud formation. Hydroperoxides, peracids, and other organic peroxides are ubiquitous and abundant components of SOAs, as summarized in table S1. Furthermore, there is laboratory evidence linking SOA-derived material to hydroxyl radical formation in aerosols and clouds. The recent work of Tong *et al*. (*25*, *26*) demonstrated an important role for Fenton-like reactions in generating OH from SOA solutions. Metal-free routes including SOA photolysis (*27*) and thermal decomposition (*25*) also produce OH with lower efficiency.

In each case, these studies have implicated organic peroxide decomposition in OH formation and suggested that only a small fraction of total SOA material participates in OH formation. This is consistent with our direct observations that OH forms efficiently for some, but not all, organic peroxides, and our laboratory experiments indicate that peracids might be especially important for this efficient OH formation. Furthermore, we estimate that organic peroxide concentrations in our ambient aerosol samples may be in the range of 10 to 100 μM following simulated cloud formation (section S9). Together, this represents considerable potential for OH production and may explain the ubiquity of the OH burst (up to 3.5 μM) in our ambient samples across different sites, seasons, and times of day.

Our observations of a rapid burst in OH formation following simulated cloud formation (when particles sampled in the field are mixed in bulk with small quantities of water) also suggest that aerosols contain a reservoir of unreacted or possibly quickly replenished precursor species. Given the chemically distinct environments of clouds (the focus here) and aerosols in terms of solute concentrations, ionic strength, and viscosity, it is certainly plausible that decomposition of organic peroxides occurs more readily in simulated cloud conditions than aerosols. In particular, highly viscous or poorly mixed aerosols would impede bimolecular Fenton-like reactions or other routes to OH production. There is some evidence that related chemistry may happen at least at the surface of particles; Wu *et al*. (*28*) found evidence of a chemical sink for PAA as it was taken up on ambient aerosols. PAA can be present at sufficiently high concentrations [up to 1 part per billion (ppb) (*29*, *30*)] and has a sufficiently high Henry’s law constant [837 M atm^−1^ (*31*)] that partitioning into cloud water, coupled with the very rapid reaction found here, may represent a major source of OH (separate from the particle-based source described here) in ambient cloud droplets.

### Importance of the OH burst

**Fig. 3:**
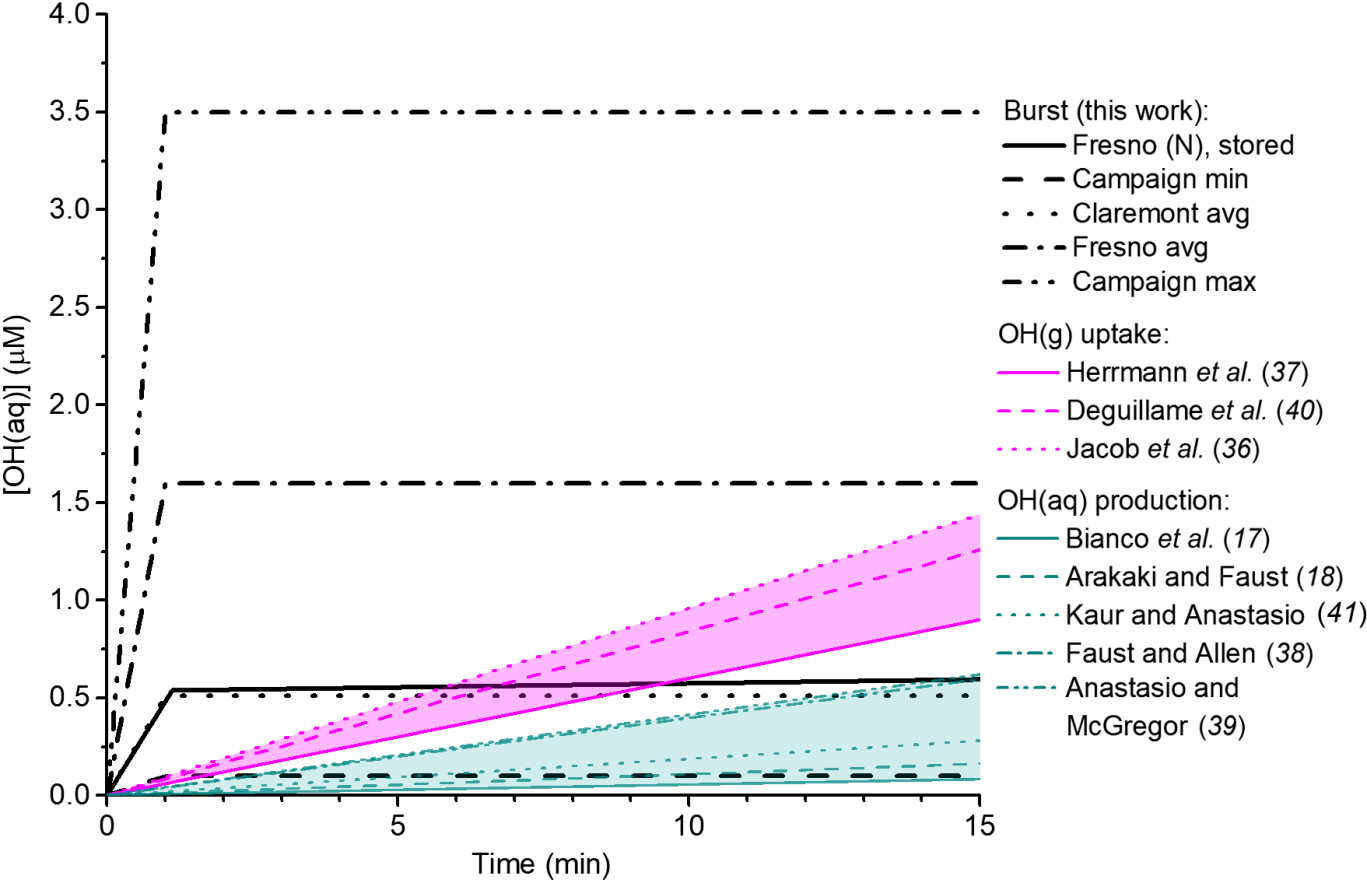
Cumulative OH(aq) formation in cloud droplets over a typical cloud droplet lifetime. Black lines: Measured production from “bursts” in this study. The solid line is a typical time-resolved burst from a Fresno stored sample (reproduced from Fig. 1). Dashed lines represent the minimum, average, and maximum cumulative concentrations from fresh samples across the campaign. Magenta lines: Uptake of OH(g) into droplets, based on estimates from three cloud chemistry models. Green lines: Measured OH(aq) production in authentic cloud and fog water samples from five previous studies (*17*, *18*, *36*–*41*).

Figure 3 shows how the cumulative OH(aq) concentration expected from other multiphase processes compares to the bursts observed in this study for typical cloud droplet lifetimes of up to 10 to 15 min (see section S10). The average [OH(aq)] observed in both Fresno and Claremont bursts exceeds that expected from all conventional aqueous phase processes [(ii) to (vii) in Introduction] based on previous measurements in authentic cloud and fog water samples. Uptake of hydroxyl radicals from the gas phase is generally thought to proceed at a rate exceeding conventional aqueous production, and Fig. 3 shows estimated uptake rates from three coupled cloud chemistry models containing detailed gas- and condensed-phase chemistry. The cumulative [OH(aq)] produced by the burst is generally comparable to these uptake estimates but, in some cases, exceeds all other OH formation processes in the droplet by up to a factor of 3 to 4 even after 10 min. This suggests that the newly identified burst process represents a major and usually dominant source of OH(aq) in cloud droplets.

The Henry’s law equilibrium concentration of OH near the surface is ~10^−12^ M. Because the concentrations of OH formed in the burst are much higher than the Henry’s law equilibrium concentration, it raises the question of escape from the particle to the gas phase. As discussed in section S11, the overwhelming majority of OH formed in the burst will be consumed by organics in the droplets and will not escape.

The comparison of all major known OH formation processes in droplets shown in Fig. 3 demonstrates that the newly discovered fast metal complex–mediated formation of OH described here could be significantly improve understanding of major uncertainties of important climate-relevant atmospheric droplet processes, such as particle oxidation and aging, DMS oxidation, or cloud condensation nuclei activity.

## METHODS

### Materials

All chemicals were purchased from Sigma-Aldrich at the highest purities available and were used as received. Water was at least 18 megaohms. Glass and Teflon containers were washed and soaked in acid baths extensively, as described by Kuang *et al*. (*32*).

### Sample collection and extraction

The collection and analysis methods are described in more detail by Kuang *et al*. (*32*) and are described only briefly here. PM2.5 or PM4 was collected on 47-mm Teflon filters (Pall Inc.) in sets of five, plus matched field blanks at three urban locations in California: Claremont, an urban/receptor site in southern California, during summer when SOA peaks; Fresno, during winter when biomass burning aerosol makes a substantial contribution to particulate matter (PM), especially at night and in the morning; and West Los Angeles, a relatively clean urban site about 8 km from the Pacific Ocean. All samples were collected from rooftops of tall campus buildings, away from direct sources. Collection times at Claremont and Fresno were 6 to 7 hours for mornings and afternoons and 13 hours for the overnight samples and were 6 to 24 hours for the West Los Angeles site. West Los Angeles samples were collected as needed to test hypotheses. The numbers of samples were 16, 16, and 17 for Claremont and 20, 22, and 18 for Fresno morning, afternoon, and overnight, respectively. Field blanks were handled in an identical manner as the samples, including loading the blank filters into the filter holders and turning on the pump for 30 s. Some analyses were performed on site, and others on filters stored for up to several years in a freezer at −4°C.

Filters were equilibrated at 30 to 70% relative humidity in room air and were cut in half with a ceramic blade and extracted in 4 ml of 18-megaohm water adjusted to pH 3.5 with sulfuric acid. For OH measurements, the extraction solution also contained 10 mM terephthalate. Assuming an average particle density of 1.3 g/cm^3^, this corresponds to volume/volume dilution factors of (26 ± 3.4 and 24 ± 9) × 10^3^ for Claremont and Fresno, respectively (range, 4700 to 180,000); Fresno ambient mass concentrations were much more variable than those at Claremont. These dilution factors fall in the range for cloud drops (*33*). A dilution of 25,000 (v/v) corresponds to a 0.4-μm-diameter particle growing to an 11.5-μm-diameter cloud drop.

Cumulative [OH] in the fresh field samples was measured every 20 min for 2 hours. Because the slope after the first 1 to 3 min is small compared to the initial burst (Fig. 1), the intercept of the 20-min resolution data corresponds to the OH formed in the initial burst.

Cumulative time-dependent OH_aq_ formation was quantified using terephthalate as a probe (*34*). Terephthalate reacts with OH_aq_ to form a fluorescent product, 2-hydroxyterephthalic acid (hTA), which is detected at λ_ex_/λ_em_ 320/420 (10 nm half-maximum) in a fluorescence cuvette in a stand-alone fluorometer (Lumina, Thermo Fisher Scientific) or also after separation with a C-18 column in a high-performance liquid chromatograph (HPLC) with a fluorescence detector (Shimadzu). The fluorometer exposes the sample to 9 s of light before each 10-ms measurement, while the HPLC records OH_aq_ formation independent of light. hTA calibration curves were generated separately for each instrument daily. Diluted aerosol samples had negligible interference from native fluorescence at 320/420, and filter blanks extracted in the same manner as samples had low or nondetectable OH_aq_ formation; all reported data have been field blank–corrected.

For the light experiments, we exposed ambient samples to 315- to 325-nm (peak width at half-maximum) light in increments of 9 s (equivalent to about 1.8 × 10^16^ photons; section S2). The photon flux was 2 × 10^15^ photons cm^−2^ nm^−1^ s^−1^ (measured as described in the Supplementary Materials), i.e., 1.8 × 10^16^ photons per exposure, delivered to a 150-μl sample in a fluorescence microcell with a 2 × 5 mm^2^ window and 1-cm pathlength (Hellma). Quantification of aqueous H_2_O_2_ was performed using an HPLC as described by Arellanes *et al*. (*35*) and in the Supplementary Materials.

## Supplementary Material

http://advances.sciencemag.org/cgi/content/full/5/5/eaav7689/DC1

Download PDF

## SUPPLEMENTARY MATERIALS

Supplementary material for this article is available at http://advances.sciencemag.org/cgi/content/full/5/5/eaav7689/DC1 Section S1. Flux of OH from the gas phase to the droplet Section S2. Photon flux determination Section S3. Further discussion of the rate of the OH burst Section S4. Relationship between OH formation and biomass burning aerosol Section S5. Quantification of H_2_O_2_ in the extraction solutions Section S6. Dependence of OH formation on the dilution factor Section S7. Potential acetyloxy or methoxy terephthalate formation and interference with fluorescence measurements Section S8. Concentration and light dependence of OH formation from PAA and Fe(II) Section S9. Peroxides in SOA Section S10. Cloud drop lifetime Section S11. Escape/consumption of OH in droplets Fig. S1. Relationship between initial OH measured on-site in fresh samples and the quantity of biomass burning aerosol. Fig. S2. Uncorrected and corrected BBA mass for all Fresno samples combined. Fig. S3. Relationship between mass-normalized OH formation and dilution factor. Fig. S4. Relationship between mass-normalized OH formation and dilution factor. Fig. S5. Concentration dependence of OH formation in the dark in aqueous pH 3.5 solution over the concentration range of 1 to 10 μM. Fig. S6. OH formation in light (320 ± 10 nm) and dark from solutions of PAA and Fe(II) at pH 3.5 about 2 min after mixing. Fig. S7. Time scale ranges for loss pathways for hydroxyl radicals in cloud droplets, assuming a 0.2- to 0.4-μm-diameter initial particle (diffusive loss to gas phase) and 5 to 35% water soluble organic carbon (WSOC) (reactive loss). Table S1. Yields and concentrations of peroxides determined in previous laboratory SOA experiments. References (*42*–*83*)
