# Supplementary material for: A light-driven burst of hydroxyl radicals dominates oxidation chemistry in newly activated cloud droplets
Source: Sci Adv. 2019 May 1;5(5):eaav7689. doi: 10.1126/sciadv.aav7689 (PMC6494489; doi:10.1126/sciadv.aav7689)
Supplement: http://advances.sciencemag.org/cgi/content/full/5/5/eaav7689/DC1 [file supp_5_5_eaav7689__index.html]

Science Advances | Science Advances

## Supplementary Materials

**This PDF file includes:**

- Section S1. Flux of OH from the gas phase to the droplet
- Section S2. Photon flux determination
- Section S3. Further discussion of the rate of the OH burst
- Section S4. Relationship between OH formation and biomass burning aerosol
- Section S5. Quantification of H2O2 in the extraction solutions
- Section S6. Dependence of OH formation on the dilution factor
- Section S7. Potential acetyloxy or methoxy terephthalate formation and interference with fluorescence measurements
- Section S8. Concentration and light dependence of OH formation from PAA and Fe(II)
- Section S9. Peroxides in SOA
- Section S10. Cloud drop lifetime
- Section S11. Escape/consumption of OH in droplets
- Fig. S1. Relationship between initial OH measured on-site in fresh samples and the quantity of biomass burning aerosol.
- Fig. S2. Uncorrected and corrected BBA mass for all Fresno samples combined.
- Fig. S3. Relationship between mass-normalized OH formation and dilution factor.
- Fig. S4. Relationship between mass-normalized OH formation and dilution factor.
- Fig. S5. Concentration dependence of OH formation in the dark in aqueous pH 3.5 solution over the concentration range of 1 to 10 μM.
- Fig. S6. OH formation in light (320 ± 10 nm) and dark from solutions of PAA and Fe(II) at pH 3.5 about 2 min after mixing.
- Fig. S7. Time scale ranges for loss pathways for hydroxyl radicals in cloud droplets, assuming a 0.2- to 0.4-μm-diameter initial particle (diffusive loss to gas phase) and 5 to 35% water soluble organic carbon (WSOC) (reactive loss).
- Table S1. Yields and concentrations of peroxides determined in previous laboratory SOA experiments.
- References (*42*–*83*)

Download PDF

**Files in this Data Supplement:**

- Adobe PDF - aav7689\_SM.pdf
