# Supplementary material for: A light-driven burst of hydroxyl radicals dominates oxidation chemistry in newly activated cloud droplets
Source: Sci Adv. 2019 May 1;5(5):eaav7689. doi: 10.1126/sciadv.aav7689 (PMC6494489; doi:10.1126/sciadv.aav7689)
Supplement: Download PDF [file aav7689_SM.pdf]

Science Advances
AAAS

advances.sciencemag.org/cgi/content/full/5/5/eaav7689/DC1

Supplementary Materials for

A light-driven burst of hydroxyl radicals dominates oxidation chemistry in newly activated cloud droplets

Suzanne E. Paulson*, Peter J. Gallimore, Xiaobi M. Kuang, Jie Rou Chen, Markus Kalberer, David H. Gonzalez

*Corresponding author. Email: paulson@atmos.ucla.edu

Published 1 May 2019, *Sci. Adv.* **5**, eaav7689 (2019)
DOI: 10.1126/sciadv.aav7689

This PDF file includes:

Section S1. Flux of OH from the gas phase to the droplet
Section S2. Photon flux determination
Section S3. Further discussion of the rate of the OH burst
Section S4. Relationship between OH formation and biomass burning aerosol
Section S5. Quantification of H_2O_2 in the extraction solutions
Section S6. Dependence of OH formation on the dilution factor
Section S7. Potential acetyloxy or methoxy terephthalate formation and interference with fluorescence measurements
Section S8. Concentration and light dependence of OH formation from PAA and Fe(II)
Section S9. Peroxides in SOA
Section S10. Cloud drop lifetime
Section S11. Escape/consumption of OH in droplets
Fig. S1. Relationship between initial OH measured on-site in fresh samples and the quantity of biomass burning aerosol.
Fig. S2. Uncorrected and corrected BBA mass for all Fresno samples combined.
Fig. S3. Relationship between mass-normalized OH formation and dilution factor.
Fig. S4. Relationship between mass-normalized OH formation and dilution factor.
Fig. S5. Concentration dependence of OH formation in the dark in aqueous pH 3.5 solution over the concentration range of 1 to 10 μM.
Fig. S6. OH formation in light (320 ± 10 nm) and dark from solutions of PAA and Fe(II) at pH 3.5 about 2 min after mixing.
Fig. S7. Time scale ranges for loss pathways for hydroxyl radicals in cloud droplets, assuming a 0.2- to 0.4-μm-diameter initial particle (diffusive loss to gas phase) and 5 to 35% water soluble organic carbon (WSOC) (reactive loss).
Table S1. Yields and concentrations of peroxides determined in previous laboratory SOA experiments.
References (*42*–*83*)

Supplementary Information

Section S1. Flux of OH from the gas phase to the droplet

Gas phase diffusion to a droplet is governed by the equation:

$$J = \frac{3D_g}{R_p^2} C \tag{S1}$$

Where J is the flux of OH to the drop, D_g is the gas phase diffusion coefficient, R_p is the drop diameter and C is the ambient gas-phase concentration.(*42*)

Section S2. Photon flux determination

The photon flux of the instrument was determined by photolyzing aqueous 0.1 – 1 mM H_2O_2 solutions with 295 – 305 nm light. OH radicals formed via H_2O_2 photolysis were measured with hydroxy terephthalate (hTA) and the instrument's photon flux at 295 - 305 was determined using absorption cross sections for H_2O_2 in aqueous solution.(*43*) This flux was extrapolated to 315 – 325 using published emission spectra for the lamp (150 W continuous Xe arc, https://www.photonics.com/Article.aspx?AID=44487).

Section S3. Further discussion of the rate of the OH burst

Aside from the limited time resolution of the measurements discussed in the main text, there are several other considerations regarding the relationship between the above rate estimates and the rate in the atmosphere. The measured value is a lower limit, as

particles loose activity as they age (Fig. 1 and discussed below); while we analyzed the fresh field samples within an hour of finishing collection, particles collected early in the sampling interval were already 5 – 13 hours old at the time of analysis.

Sunlight contains a maximum of ~5.4×10^{15} photons cm^{-2} s^{-1} in the 296 – 350 nm wavelength range.(*13*) The photon flux in our experiments, 2×10^{16} photons cm^{-2} s^{-1} over the 315 – 325 nm wavelength interval is somewhat larger than the photon flux from sunlight. However, the ratio of the cross section to volume of 1 – 20 μM droplets is > 10^3 × larger than in our cuvette-based measurements, thus the light-driven OH formation chemistry in cloud and fog droplets could be two orders of magnitude faster than observed in our measurements, suggesting the OH burst might have a rate of ~ 1 – 30 × 10^{-8} M s^{-1}. Finally, the rate of formation may also be affected by dissolution kinetics of the particles.

Section S4. Relationship between OH formation and biomass burning aerosol

Figure S1 shows the relationship between the biomass burning aerosol (BBA) content and OH formation. These data have not been normalized to aerosol mass (i.e. divided by the aerosol mass) or to the dilution factor as have Figs. 1a and b, but rather are plotted as a function of the absolute quantity of BBA. Mass and biomass burning content are obviously linked (fig. S2), so controlling for mass removes some of the effect of the material from biomass burning. The mass of biomass burning is much more

strongly correlated to the initial OH formation than either the fraction of biomass burning aerosol in the particles or PM mass.

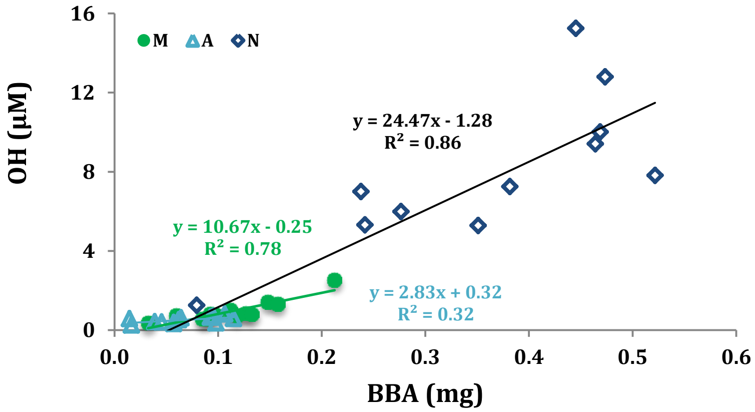

Fig. S1. Relationship between initial OH measured on-site in fresh samples and the quantity of biomass burning aerosol. M = morning samples, A = afternoon samples, and N = overnight samples.

Quantification of the contribution of BBA on PTFE filters was accomplished using optical absorption at 370 nm and 880 nm respectively. Measurements where performed with an OT21 duel wavelength optical transmissometer (Magee Scientific Corporation). A quartz diffuser backing (Pallflex Fiberfilm) was placed under each PTFE filter to provide an even distribution of light to the detector; care was taken to ensure the filter lay flat against the quartz diffuser. Light absorption at 880 nm is proportional to the concentration of absorbing aerosol,(*44*, *45*) expressed with the following equation:

$$[\mathrm{BBA}] = \frac{b_{ATN}}{\sigma(\lambda)_{ATN}} = \frac{\mathrm{ATN}\times \mathrm{A}}{\sigma(\lambda)\times \mathrm{V}} = \frac{100\ \mathrm{Ln}\left(\frac{\mathrm{Io}}{\mathrm{I}}\right)\times \mathrm{A}}{\sigma(\lambda)\times \mathrm{V}} \qquad \text{(SI Eqn. 1)}$$

Where b_{ATN} is the attenuation coefficient, $\sigma(\lambda)_{ATN}$ (m^2/g) is the specific attenuation cross section, A (m^2) is the filter area, V (m^3) is the volume of air sampled, ATN is the attenuation, I and Io are light transmitted through a sample and blank respectively. The concentration of biomass burning aerosol [BBA] is also calculated from equation 1 using values at 370 nm. $\sigma(\lambda)_{ATN}$ (m^2/g), the attenuation cross section at a specific wavelength, represents a substantial source of uncertainty in the determination of BBA via optical methods. Literature values for sigma span a wide range of values, 2-25 m^2/g (*46-49*); differences can be attributed to the aerosol source and composition, aerosol age, different filter materials, correction formulas and various optical and thermal optical techniques to determine Black/elemental carbon.(*46, 47, 50*) For this work we selected $\sigma_{ATN}(370)$ of 9.6 $m^2\ g^{-1}$ and $\sigma_{ATN}(880)$ of 4.2 $m^2\ g^{-1}$ used to convert ATN to a BBA concentration. These values were empirically determined by the EPA for PTFE filters from FRM-CSN sites (*51*). The sites include urban emissions sites and biomass burning emission sites, with σ_{ATN} only varying ± 10% among sites. These values also closely resemble the attenuation cross-sections determined for the Fresno supersite (Watson et al., 2010) which closely resembled our sampling site in Fresno.

Shadowing Correction

Filter-based optical methods are complicated by the well-known problem that increased filter loading results in shadowing and other optical effects. As a result, direct measurements of ATN tend to underestimate the true BC concentration.(*52-54*) The

same problem is expected for brown carbon at 370 nm. The particle loading effect can be avoided by minimally loading the filters,(*55*) however since our filters were used primarily for chemical assays, we had higher loadings. Several algorithms have been formulated to correct for nonlinearities due to particle loading.(*52, 56-58*) We considered the formulations suggested by Virkkula et al.(*57*) and Jimenez et al.(*56*) as their methods did not require the aerosol scattering coefficients, which we did not have. The two algorithms produced very similar results. Here we use the correction developed by Jimenez et al.(*56*) using diesel exhaust. It uses an empirically derived an expression for K(ATN), an attenuation-dependent correction factor to correct for the underestimation of BC and aerosol absorption at 880 and 370 nm:

$$K(ATN) = a + b * \exp(-ATN/100) = a + b * T \quad \text{(SI 2)}$$

$$[BBA]_c = \frac{bATN}{\sigma ATN * K(ATN)} \quad \text{(SI 3)}$$

Where a and b are linear regression coefficients, T is the filter transmission and $[BBA]_c$ is the corrected BBA concentration. At 880 nm for the correction of BC, a = 0.13 and b = 0.88. For the correction of BBA at 370 nm, a = 0.38 and b = 0.67.

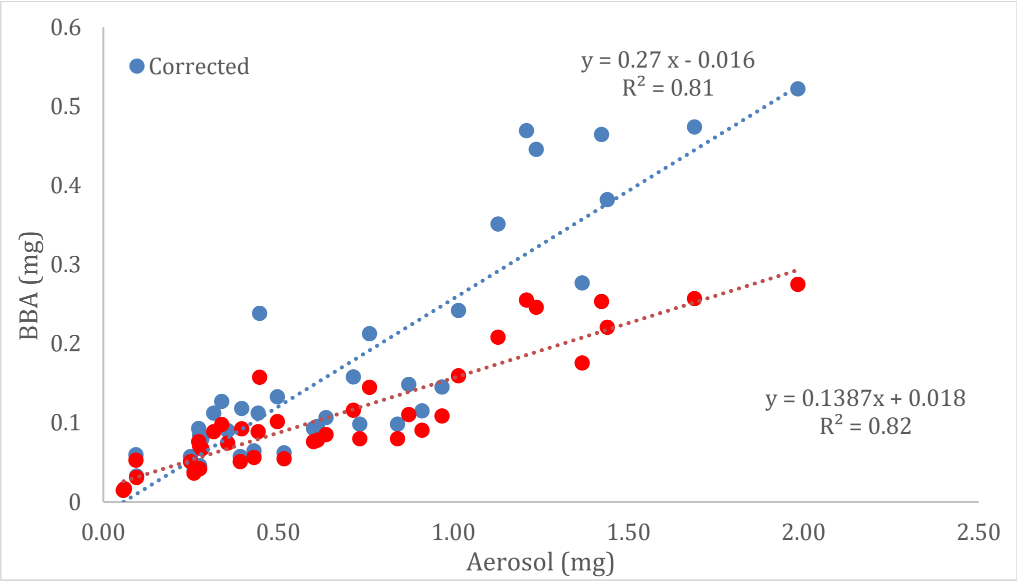

Fig. S2. Uncorrected and corrected BBA mass for all Fresno samples combined.

Section S5. Quantification of H_2O_2 in the extraction solutions

100 µL aliquots of the filter extracts were analyzed for H_2O_2 using a High Performance Liquid Chromatography (HPLC) (Shimadzu).(*35*) The eluent system, consisting of pH 3.5 solution adjusted with 0.1 N sulfuric acid and 0.1 mM EDTA, was delivered by an isocratic pump at 0.6 mL/min. A C18 guard column was installed to remove any impurities. H_2O_2 was eluted at 0.6 mL/min, after which it was mixed with a fluorescent reagent containing horseradish peroxidase and *para*-hydroxyphenyl acetic acid (POHPAA). The peroxidase enzyme catalyzes the reaction between H_2O_2 and POHPAA, forming a fluorescent dimer, which is detected with a fluorescence detector at the

excitation/emission wavelengths of 300/420 nm. A 30% ammonium hydroxide solution was mixed with the dimer prior entering the detector to increase fluorescence signal. Calibrations were performed weekly or when the HPLC conditions were changed. Standards ranging from 10^{-8} to 10^{-6} M were prepared from 0.3% stock solution, which was titrated with sodium thiosulfate to determine the stock solution concentration (about 0.1 M). The detection limit for this method is 10 nM.

H_2O_2 measured in the dark aerosol extraction solutions was initially below the detection limit (~2 nM), and it built up over the first two hours to concentrations in the 30 – 1000 nM range (not shown) as has been observed earlier.(*35, 59, 60*)

Section S6. Dependence of OH formation on the dilution factor

Figure S3 shows the initial OH formation normalized to mass, plotted vs. the dilution factor. Generally, OH formation increases with increasing biomass burning particle mass (fig. S1). Consistent with this, in our assay, in which the extraction volume was held constant, it decreases with increasing dilution factor. To examine the effect of dilution (or concentration) itself, we first normalize the OH formation to the mass in the sample. Over most of the range of dilution tested here (1 – 5) × 10^4, and for most samples, the initial OH is mostly weakly- or un-correlated with the dilution factor (fig. S3, Claremont morning, afternoon and night and Fresno morning and afternoon). Claremont samples had weak positive (overnight) or not significant (Claremont

morning and afternoon) slopes. For the Fresno (fig. S4) morning and afternoon samples, high dilution factors above (1 – 2) × 10^5 had somewhat higher mass-normalized OH formation, although we have only 4 samples in this range, and all were collected during or immediately after rain events. Excluding these points, there was no significant trend for the morning samples ($r^2 = 0.04$), and a weak, small positive trend for the afternoon samples ($r^2 = 0.25$). Apart from the rain events, the Fresno night samples had high masses, high contributions from biomass burning aerosol and low dilution factors, mostly 5,000 – 10,000. For these samples, the OH burst vs. dilution factor had a higher slope; the mass-normalized OH formation approximately doubles as the dilution increased from 4500 to 10000, but the data are also scattered ($r^2 = 0.22$). Additionally, one sample collected during the rain event does not fit this trend (fig. S4). Overall, the trends imply that our measurements containing high levels of biomass burning aerosol may somewhat underestimate the strength of the OH burst from these particles (fig. S4).

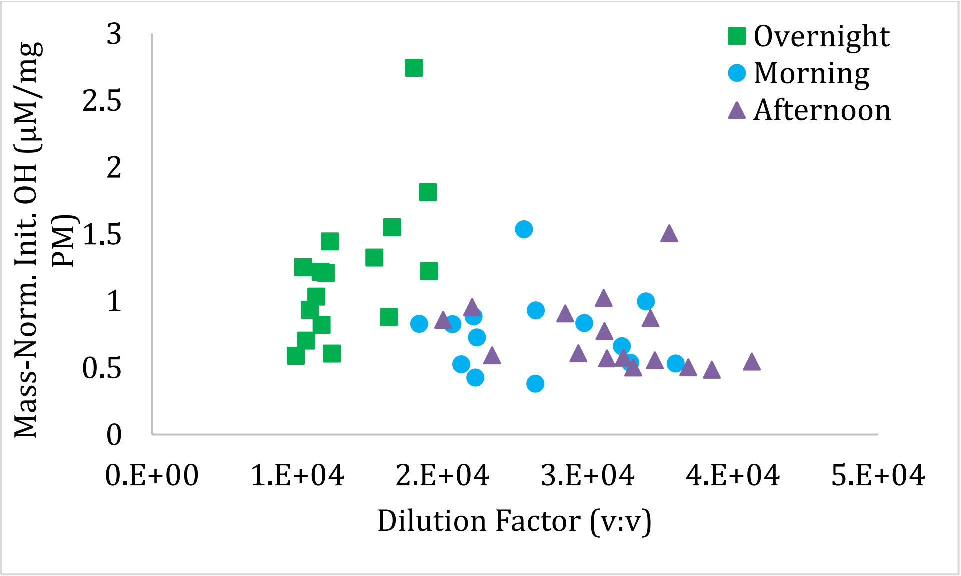

Fig. S3. Relationship between mass-normalized OH formation and dilution factor. Green squares, blue diamonds and orange triangles show Claremont night, morning and afternoon samples respectively.

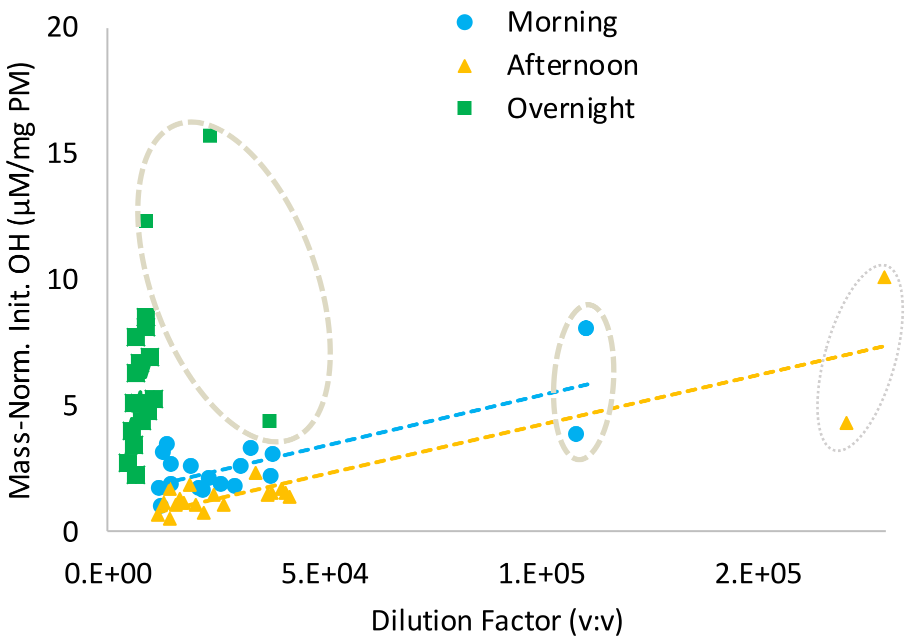

Fig. S4. Relationship between mass-normalized OH formation and dilution factor. Green and dark blue squares, light blue circles and orange triangles show Fresno night, morning and afternoon samples respectively. Circled data points all correspond to samples collected during or immediately after a rain event. The circled green points were not included in the regression for the overnight data.

Section S7. Potential acetyloxy or methoxy terephthalate formation and interference with fluorescence measurements

The terephthalate probe is widely accepted to be specific for hydroxyl radicals,(*61, 62*) however here we explore the possibility that the probe has an interference in the PAA system. Breakdown of peracetic acid may produce both OH radicals and acetyloxyl radicals, $CH_3C(=O)O\cdot$. Loss of CO_2 from the acetyloxyl radicals is exothermic and is generally accepted to be rapid (μs or faster) in solution.(*63, 64*) The remaining methyl radical is expected to be converted to the methyl peroxyl radical, $CH_3OO\cdot$ upon addition of oxygen. Some of this might be converted to the methoxy radical, $CH_3O\cdot$. Methoxy radicals may react fairly rapidly with O_2 (estimated rate constant 1.2×10^6 M^{-1} s^{-1})(*65*) to form formaldehyde and $HO_2\cdot$ or rearrange to the $\cdot CH_2OH$ radical(*66*) (estimated rate constant 1×10^6 s^{-1}).(*65*) Literature suggests that alkoxy radicals prefer abstraction from rather than addition to allylic bonds, so formation of methoxy terephthalate is not expected.(*67, 68*) As the fluorescence arises from a $\pi \rightarrow \pi^*$ transition involving the oxygen substituent on the ring, addition of $\cdot CH_2OH$ (probably also unlikely) would not be expected to produce a fluorescent product.

Section S8. Concentration and light dependence of OH formation from PAA and Fe(II)

Figure S5 shows the dependence of OH formation on the concentration of Fe(II) and PAA in the dark, with the two reactants in a constant ratio. The molar yield decreases with increasing concentration, a phenomenon that may be the result by formation of dimers or higher order complexes of iron and its complexes, both with PAA and OH^-.

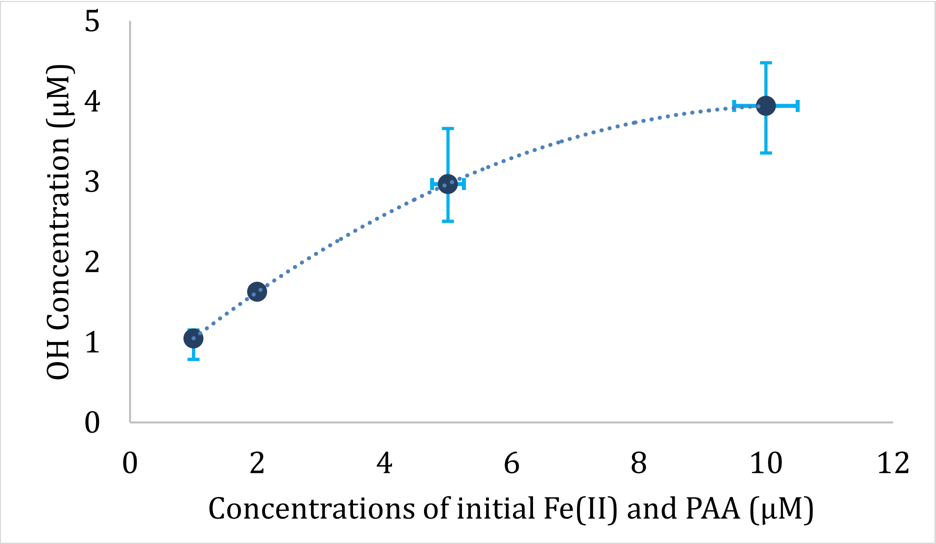

Fig. S5. Concentration dependence of OH formation in the dark in aqueous pH 3.5 solution over the concentration range of 1 to 10 μM. A quadratic polynomial is shown to guide the eye.

Figure S6 shows OH formation from solutions with Fe(II) at 1 μM and PAA at 1, 5 and 10 μM. While the ratio of the two is not constant, formation in light is in all cases at least double formation in the dark. Additional PAA increases OH formation up to a point, after which it no longer increases OH formation. In the dark, formation of OH is roughly stoichiometric with Fe(II), consistent with a Fenton-like reaction (R1).

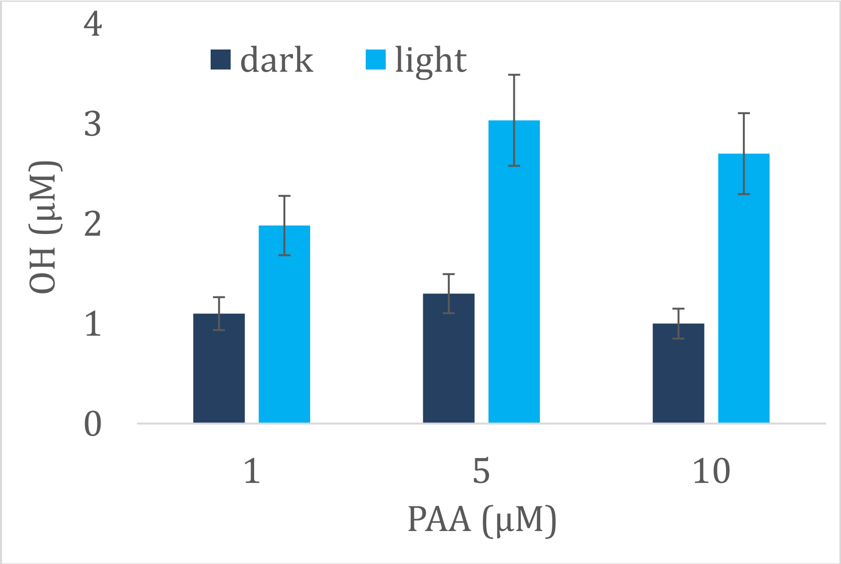

Fig. S6. OH formation in light (320 ± 10 nm) and dark from solutions of PAA and Fe(II) at pH 3.5 about 2 min after mixing. Initial Fe(II) was 1 μM for all measurements.

Section S9. Peroxides in SOA

The yields of organic peroxides in SOA laboratory studies vary considerably depending on the precursor and formation conditions; a summary is provided in table S1.

Table S1. Yields and concentrations of peroxides determined in previous laboratory SOA experiments. NB some of these use iodometric techniques also in principle sensitive to H_2O_2.

Study	System	Relative yield (%)	Conc. (Aerosol) (nmol/μg)	Notes
**Docherty, Wu, Lim and Ziemann (*69*)**	α-pinene + ozone	47		Assumes aerosol density = 1. 1.3 gives 36% yield.
	β-pinene + ozone	85		As above
**Ziemann (*70*)**	Oleic acid + ozone	68		
**Surratt, Murphy, Kroll, Ng, Hildebrandt, Sorooshian, Szmigielski, Vermeylen, Maenhaut, Claeys, Flagan and Seinfeld (*71*)**	Isoprene photo-oxidation	61; 25-30		Unseeded; seeded
**Nguyen, Bateman, Bones, Nizkorodov, Laskin and Laskin (72)**	Isoprene + ozone		1 ± 0.1	
**Bateman, Nizkorodov, Laskin and Laskin (*73*)**	Limonene + ozone	2		
**Sato, Takami, Kato, Seta, Fujitani, Hikida, Shimono and Imamura (*74*)**	1,3,5-TMB photo-oxidation	12 ± 8		
**Mertes, Pfaffenberger, Dommen, Kalberer and**	α-pinene photo-oxidation	12-34; 5-6		Low NO_x; High NO_x

**Baltensperger (*75*)**				
**Epstein, Blair and Nizkorodov (*76*)**	α-pinene + ozone	22 ± 5		
**Badali, Zhou, Aljawhary, Antiñolo, Chen, Lok, Mungall, Wong, Zhao and Abbatt (*27*)**	α-pinene + ozone	0.5		
**Krapf, Haddad, Bruns, Krapf, Haddad, Bruns, Molteni and Daellenbach (*77*)**	α-pinene + OH	9	1-2	
**Jiang, Jang and Yu (*78*)**	Toluene photo-oxidation		3-6	NO_x dependent
	Isoprene photo-oxidation		1-3	NO_x dependent
**Gallimore, Mahon, Wragg, Fuller, Giorio, Kourtchev and Kalberer (*79*)**	Limonene ozonolysis		0.4	Concentration of "stable ROS"

Organic peroxide concentrations have been measured as 1-6 nmol/µg aerosol from SOA across biogenic and anthropogenic VOC precursors.(*72*, *77*, *78*) In the absence of ambient data, we use this range to estimate initial concentrations of organic peroxides in our simulated cloud water. Assuming an aerosol density of 1.3 g/cm^3, an organic aerosol mass fraction of 0.3 and a dilution factor of 25,000, we estimate organic peroxide concentrations in the range 15-90 µM may be present, which represents a considerable reservoir for OH production, consistent with providing sufficient precursors for the observed OH burst of up to 3.5 µM (Fig. 1b). A few ambient studied have quantified organic peroxides and peroxy acids, primarily methyl hydroperoxide and peroxy acetic

acid in the gas phase. They found concentrations typically in the range 0.1-1 ppb (*30*) (*29, 80*) with Phillips et al. (*29*) reporting total [ROOH(g)] up to 2 ppb.

Section S10. Cloud drop lifetime

We assume a cloud droplet lifetime of 15 minutes. The lifetime of cloud drops has not been extensively characterized and is clearly variable, but several studies suggest 15 minutes is a reasonable mid-range estimate. The lifetime of several types of cumulus clouds ranges from ~10 – 35 minutes,(*81*) but this represents an upper limit for the average lifetime of a droplet, as clouds are dynamic, continuously condensing/evaporating at their edges, and in some cases precipitating. Modeling studies often consider 10 - 15 minutes.(*6, 82*)

Section S11. Escape/consumption of OH in droplets

The Henry's law equilibrium concentration of OH near the surface is ~10^{-12} M, but concentrations quickly decay away from the surface of the drop due to reactions with organics.(*6*) The OH burst (in addition to other known sources of OH) inside the droplets provide an additional source of OH to the bulk. Because the concentrations of OH formed in the burst are much higher than the Henry's law equilibrium concentration (SI), it raises the question of escape from the particle to the gas phase. Figure S7 shows the characteristic time scales for OH loss via liquid phase diffusion to the droplet surface and loss via reaction with organics in the drop. Diffusive loss depends on droplet diameter; bounds for particles with initial diameters of 0.2 and 0.4

μm are shown. Reactive loss depends only on the fraction of WSOC in the initial particle and the dilution factor. The bounds indicate 5 and 35% WSOC, 20 g/mol C in WSOC and 3.8×10^8 M s^{-1}.(*83*) Figure S7 shows that despite the OH burst generating sufficient OH to form a highly supersaturated solution, organics within the droplets are sufficiently abundant to prevent its escape to the gas phase.

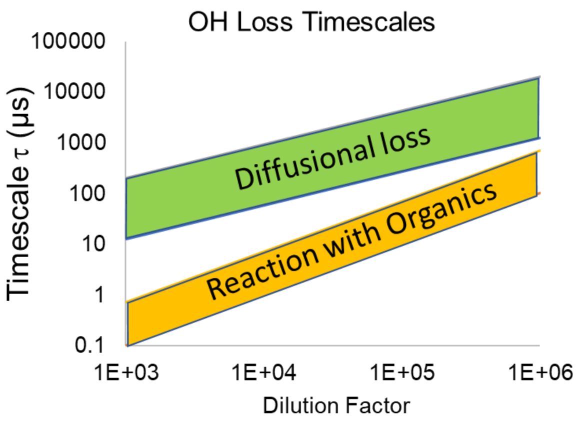

Fig. S7. Time scale ranges for loss pathways for hydroxyl radicals in cloud droplets, assuming a 0.2- to 0.4-μm-diameter initial particle (diffusive loss to gas phase) and 5 to 35% water soluble organic carbon (WSOC) (reactive loss).
